# Special issue on the role of extracellular vesicles in human diseases

**DOI:** 10.1038/s12276-019-0208-4

**Published:** 2019-03-15

**Authors:** Yong Song Gho, Jaewook Lee

**Affiliations:** Department of Life Sciences, Pohang University of Science and Technology (POSTECH), Pohang, 37673 Republic of Korea

Almost all cells on earth release vesicles, which are nano-sized enclosed structures with a lipid bilayer, into the extracellular environment^1–4^. Many different terms, such as exosomes and microvesicles, have been used for such vesicles, which can collectively be called extracellular vesicles^5^. Extracellular vesicles contain diverse bioactive components, such as proteins, lipids, nucleic acids, and metabolites^6,7^, that represent a subset of cellular components, implying the existence of cargo-specific sorting mechanisms^8^. These vesicles are present in biological fluids and environments and have been proposed to play diverse pathophysiological roles, such as immune modulation, angiogenesis, tissue repair, and tumor metastasis^9,10^. Extracellular vesicle-mediated intercellular and interkingdom communication are evolutionarily conserved phenomena^1–4^. The field of extracellular vesicle research is intensively expanding, as indicated by a rapid increase in the number of extracellular vesicle-related publications collected in EVpedia (http://evpedia.info), a community web portal for extracellular vesicle research^11,12^. Current research has suggested using extracellular vesicles as next-generation targets for diagnostics and therapeutics^13,14^. However, systematic and comprehensive studies based on emergent properties of these vesicles rather than reductionist approaches are essential for understanding the complexity of extracellular vesicle-mediated intercellular and interkingdom communication^1–4^. The aim of this Special Issue of *Experimental & Molecular Medicine*, which is entitled “Role of Extracellular Vesicles in Human Diseases”, is to highlight the significance of extracellular vesicles in human diseases.

In this Special Issue, we present five review articles that provide insights into current knowledge regarding the roles of extracellular vesicles in human diseases. Kim et al. introduce proteome profiling of extracellular vesicles from different cancers using mass spectrometry-based proteomic approaches^15^. They specifically note the use of extracellular vesicle-associated proteins as predictive cancer biomarkers and the importance of enriched proteins in cancer-associated extracellular vesicles in cancer development and progression; thus, these vesicle-enriched proteins could be potential therapeutic targets. Buzás et al. present an overview of systems biology approaches to investigate the roles of extracellular vesicles in human diseases^16^. They provide examples of how such approaches can be used to identify correlations between genes involved in extracellular vesicle biogenesis and diseases. Ochiya and Kosaka et al. introduce current studies regarding extracellular vesicles as biomarkers for cancer diagnosis based on the biology of extracellular vesicles in cancer development^17^. They also introduce several technologies to detect extracellular vesicles to enable the use of extracellular vesicle-associated molecules for cancer diagnosis. Camussi et al. discuss the roles of extracellular vesicles in onco-nephrology^18^. They especially emphasize the role of renal cancer stem cell-derived extracellular vesicles in favoring tumor aggressiveness, angiogenesis, and immune modulation. Schiffelers and Vader et al. review current knowledge regarding the targeting, internalization, and intracellular trafficking of extracellular vesicles^19^. They also provide engineering approaches that impact the circulation kinetics, bio-distribution, targeting, and internalization of extracellular vesicles.

We hope the review articles presented in this Special Issue of *Experimental & Molecular Medicine* shed light on the significance of extracellular vesicles in human diseases. Steady research on the components and functions of extracellular vesicles would facilitate the use of extracellular vesicles in diagnostics and therapeutics. Finally, we appreciate the efforts of all contributors to this Special Issue.
